# Pathology-Guided Transcriptomic Profiling of Prostate Cancer Identifies Grade-Associated Proliferative and Immune Signatures in a MENA Cohort

**DOI:** 10.3390/cancers18182898

**Published:** 2026-09-08

**Authors:** Ranyah Al-Hakm, Alaa Muayad Altaie, Reem Sami Alhamidi, Anania Boghossian, Nival Ali, Alaa Mohamed Hamad, Eman Sheta, Nagwa Mashali, Riyad Bendardaf, Timo Gemoll, Iman M. Talaat, Rifat Hamoudi

**Affiliations:** 1 Research Institute of Medical and Health Sciences, University of Sharjah, Sharjah P.O. Box 27272, United Arab Emirates; u22104866@sharjah.ac.ae (R.A.-H.);; 2 Clinical Sciences Department, College of Medicine, University of Sharjah, Sharjah P.O. Box 27272, United Arab Emirates; 3 Section for Translational Surgical Oncology & Biobanking, Department of Surgery, University of Luebeck, Ratzeburger Allee 160, 23538 Luebeck, Germany; 4 Center of Excellence for Precision Medicine, Research Institute of Medical and Health Sciences, University of Sharjah, Sharjah P.O. Box 27272, United Arab Emirates; 5 College of Health Sciences, Abu Dhabi University, Abu Dhabi P.O. Box 59911, United Arab Emirates; 6 Pathology Department, Faculty of Medicine, Alexandria University, Alexandria P.O. Box 21131, Egypt; 7 Oncology Unit, University Hospital Sharjah, Sharjah P.O. Box 72772, United Arab Emirates; 8 Center of Excellence for Cancer Research, Research Institute of Medical and Health Sciences, University of Sharjah, Sharjah P.O. Box 27272, United Arab Emirates; 9 BIMAI-Lab, Biomedically Informed Artificial Intelligence Laboratory, University of Sharjah, Sharjah P.O. Box 27272, United Arab Emirates; 10 ASPIRE Precision Medicine Research Institute, Abu Dhabi, University of Sharjah, Sharjah P.O. Box 27272, United Arab Emirates; 11 Division of Surgery and Interventional Science, University College London, London WC1E 6TB, UK

**Keywords:** prostate cancer, Gleason score, transcriptomic profiling, RNA sequencing, NF-κB signaling

## Abstract

Background: Prostate cancer (PC) is a heterogeneous disease in which the histopathological grade does not always reflect underlying biological behavior. While transcriptomic studies have provided insight into tumor biology, grade-associated molecular changes remain incompletely defined, particularly in underrepresented populations such as those from the Middle East and North Africa (MENA) region. Methods: In this study, we used a pathology-guided transcriptomic approach to examine gene expression patterns across PC grades in a MENA cohort. RNA sequencing was performed on formalin-fixed paraffin-embedded (FFPE) tissues, including benign prostatic hyperplasia (BPH, *n* = 7), low-grade tumors (Gleason 6–7 [3 + 4]; LG; *n* = 7), and high-grade tumors (Gleason 7 [4 + 3]–10; HG; *n* = 7). Differential expressions, pathway-level enrichment analyses and the estimation of immune cell composition were carried out, followed by comparison with publicly available datasets (TCGA-PRAD, *n* = 496), including LG tumors (*n* = 292) and HG tumors (*n* = 204), using the GEPIA2 and UALCAN platforms. Selected genes were further assessed using qRT-PCR in an independent set of samples (*n* = 21). Representative immunohistochemical images from the Human Protein Atlas were reviewed to provide descriptive protein-level context across tumor grades. Results: Transcriptomic comparisons of tumor samples with benign controls identified a set of shared transcriptional changes present in both low- and high-grade disease. Among these, SLC29A2 and IL2RA showed consistent upregulation across tumor grades and similar expression trends in external datasets. Direct comparison between HG and LG tumors revealed distinct transcriptional profiles with patterns indicative of increased proliferative activity and altered immune-related signaling in higher-grade disease. Pathway-level analyses showed the enrichment of cell-cycle and metabolic gene signatures in HG tumors, whereas inflammatory and NF-κB-associated transcriptional signatures showed a comparatively reduced expression. Additional genes, including *AAMDC, ASAH2, TNFRSF1B, NFKBIL1,* and *NFKBIZ*, were associated with these grade-dependent differences. qRT-PCR findings were generally consistent with the RNA-seq results for selected targets. Conclusions: This study describes transcriptional patterns associated with the PC grade in a MENA cohort using a pathology-guided framework. The findings highlight candidate genes associated with tumor presence and grade progression and provide a foundation for further investigation in larger, well-annotated cohorts, particularly in populations that remain underrepresented in transcriptomic studies.

## Simple Summary

Prostate cancer is a heterogeneous disease, and molecular markers that reflect tumor grade progression remain limited. In this study, we analyzed gene expression changes across benign, low-grade, and high-grade prostate cancer tissues using a pathology-guided transcriptomic approach in a Middle East and North Africa cohort. We identified shared transcriptional alterations associated with tumor presence and distinct gene expression patterns linked to higher tumor grade. Several candidate genes were supported by external datasets and validated by qRT-PCR in independent prostate specimens. These findings provide grade-associated molecular insights into prostate cancer progression and highlight potential biomarkers for further pathological and clinical investigation.

## 1. Introduction

Prostate cancer (PC) remains one of the most frequently diagnosed malignancies and a leading cause of cancer-related mortality among men worldwide [1,2]. The global burden of PC continues to rise, with projections indicating substantial increases in both incidence and mortality over the coming decades [3]. Although historically more prevalent in Western populations, the incidence of PC is rising across developing regions, including the Middle East and North Africa (MENA), where epidemiological data indicate a growing clinical and public health impact [4,5]. In 2022, an estimated 53,112 new cases and 20,034 deaths were reported among males in the MENA region, highlighting the increasing burden of disease within this population [6]. In countries such as the United Arab Emirates, PC is among the most prevalent cancers among men, underscoring the importance of improving disease characterization and risk stratification within regional populations [3].

Despite advances in the large-scale transcriptomic and genomic profiling of PC, patients from the MENA region remain underrepresented in publicly available datasets, limiting the generalizability of current molecular classifications. Given the potential influence of population-specific genetic background, environmental exposures, and healthcare variability, region-specific investigations are essential to better define transcriptional programs associated with disease progression. Region-specific investigations are essential to advance the molecular characterization of PC and improve its clinical stratification in underrepresented populations.

A defining feature of PC is its marked biological and clinical heterogeneity [7]. While many tumors follow an indolent course, a subset progresses to aggressive, locally advanced, or metastatic disease [8,9]. This variability presents a major challenge in clinical management, as current diagnostic and prognostic tools remain insufficient to reliably distinguish indolent tumors from those with high progression potential. Prostate-specific antigen (PSA)-based screening, although widely used, lacks specificity and is associated with overdiagnosis and overtreatment [10]. Reliable biomarkers capable of distinguishing indolent from aggressive disease at diagnosis remain lacking [11].

Histopathological evaluation, particularly the Gleason grading system refined by the International Society of Urological Pathology (ISUP), remains the cornerstone of clinical risk stratification [12,13,14]. However, tumors with similar Gleason scores may exhibit markedly different clinical behavior, reflecting underlying molecular diversity. For example, tumors with a Gleason score 7 (3 + 4) often differ biologically and clinically from those with a Gleason score 7 (4 + 3) despite sharing the same composite score. These observations highlight the need for molecular markers that better capture disease biology and complement existing pathological assessment. At the molecular level, PC progression is driven by complex alterations in transcriptional programs regulating proliferation, metabolism, immune signaling, and tumor–microenvironment (TME) interactions [15,16]. Advances in high-throughput sequencing have established RNA sequencing (RNA-seq) as a robust platform for the genome-wide characterization of gene expression and tumor-associated transcriptional changes [17]. Transcriptomic profiling has therefore been increasingly applied to investigate molecular heterogeneity and identify clinically relevant signatures in PC [18,19,20]. In addition, recent methodological advances have enabled reliable transcriptomic analysis of formalin-fixed paraffin-embedded (FFPE) tissues despite RNA degradation, thereby expanding opportunities for translational research using archived clinical specimens [21,22,23].

Despite these advances, most large-scale transcriptomic and genomic studies of PC have been conducted in Western populations, with limited representation of patients from the MENA region. This underrepresentation may limit the generalizability of existing molecular insights, given the potential differences in genetic background, environmental exposures, and clinical presentation. Moreover, while previous transcriptomic studies have primarily focused on comparisons between malignant and benign tissues, fewer investigations have specifically examined transcriptional differences across histopathological grades within clinically stratified cohorts. As a result, the molecular basis of grade-associated disease progression in MENA populations remains insufficiently characterized, representing a critical gap in current knowledge.

In this context, the present study applies a pathology-guided transcriptomic approach to characterize gene expression patterns associated with Gleason grade progression in a MENA cohort. Prostate tissues, including benign prostatic hyperplasia (BPH), low-grade (LG), and high-grade (HG) tumors, were analyzed using RNA sequencing, followed by pathway-level enrichment analyses, immune composition estimation, and validation in independent datasets. Candidate genes identified from transcriptomic analyses were further validated using qRT-PCR in independent FFPE prostate tissues. By integrating histopathological stratification with transcriptomic profiling, this study aims to characterize grade-associated molecular signatures and provide biologically informed insights into PC progression in an underrepresented population.

## 2. Materials and Methods

**Figure 1:**
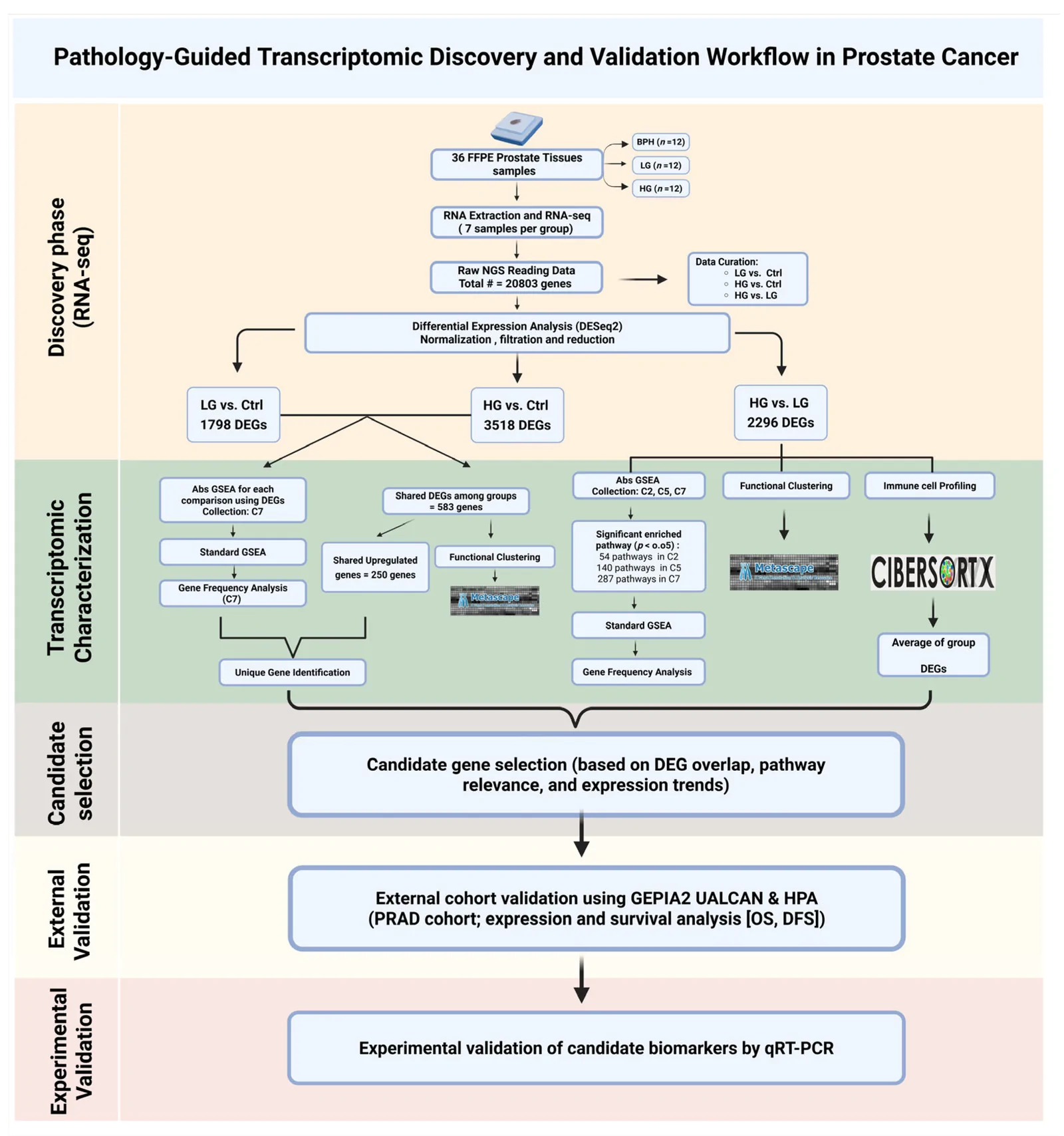
Schematic representation of the study design and bioinformatics workflow used to define molecular signatures and identify candidate biomarkers across PC grades. FFPE prostate tissues comprising BPH, LG, and HG samples were subjected to RNA sequencing to identify grade-associated transcriptional changes. Differential expression analysis was followed by functional characterization, including GSEA, functional clustering, and immune cell profiling, to prioritize candidate genes. Candidate selection was based on DEG overlap, pathway relevance, and expression trends. Selected candidates were subsequently validated using independent external cohorts (GEPIA2, UALCAN, and HPA) and experimentally confirmed by qRT-PCR. Abbreviations: FFPE: formalin-fixed paraffin-embedded; BPH: benign prostatic hyperplasia, used as a control in this study; LG: low grade; HG: high grade; GSEA: gene set enrichment analysis; HPA: Human Protein Atlas; DFS: disease-free survival; OS: overall survival; and PRAD: prostate adenocarcinoma. # indicates the total number. Created in BioRender. Alhakm, R. (2026) https://BioRender.com/mct80kp (accessed on 25 April 2026).

The schematic diagram of the study design and workflow is given in Figure 1.

### 2.1. FFPE Specimens from Prostate Tissues

This study involved a total cohort of thirty-six prostate tissue samples. Of these, twenty-one samples (seven BPH and fourteen PC samples) were used for transcriptomic analysis. An additional fifteen independent samples were included for qRT-PCR validation, along with six overlapping samples (two from each group) incorporated for validation purposes.

The samples were provided as FFPE tissue specimens. The cohort comprised seventeen samples from the Middle East and nineteen samples from North Africa. Ethical approval was obtained from two independent institutions: the Hospital Ethics and Research Committee, University Hospital Sharjah, UAE (Ref. No.: UHS-HERC-176-16092049), and the Faculty of Medicine, Alexandria University, Egypt (Ref. No.: 0306768). All procedures were conducted in accordance with the ethical standards outlined in the Helsinki Declaration and the Belmont Report.

Classification according to the Gleason score [12,13] was derived from patients’ pathology reports and reviewed by expert histopathologists. BPH samples served as controls in this study. PC samples were classified into two histopathological subtypes, HG and LG. The inclusion criteria were an HG grading of 7 (4 + 3), 8, 9, or 10, and an LG grading of 7 (3 + 4) or 6. The patient’s clinicopathological data, including age, tumor subtype, PSA levels and Gleason score, are presented in Table S1.

FFPE tissue sections were reviewed by a pathologist, and regions with ≥70% tumor epithelial cellularity were selected for RNA extraction to reduce stromal and non-tumor cell admixture. This approach was applied to enrich tumor-derived transcriptional signals. The cohort size was supported by sample size estimation for targeted gene expression analyses [24] using the parameters delta = 15, sigma = 10, power = 0.9, and significance level (*p*-value) = 0.05. The power calculation indicated that the minimum number of samples required for detecting differences in targeted gene expression analyses is seven. Accordingly, RNA-seq was conducted within an exploratory hypothesis-generating framework.

### 2.2. RNA Extraction from FFPE Samples

RNA was extracted for whole-transcriptome sequencing. Briefly, the 21 FFPE tissue specimens were individually cut into three sequential sections of 3 μm thickness. RNA was then extracted using the RecoverAll^TM^ kit as per the manufacturer’s protocol (Thermo Fisher Scientific, Waltham, MA, USA). Furthermore, genomic DNA was removed from all RNA samples through treatment of extracted RNA with TURBO DNAase-free^TM^ kit (Invitrogen, Carlsbad, CA, USA). RNA concentration and purity were assessed using a NanoDrop™ 2000 spectrophotometer (Thermo Fisher Scientific, Waltham, MA, USA). Samples selected for RT-qPCR validation exhibited A260/A280 ratios ranging from 1.91 to 2.0 and A260/A230 ratios greater than 1.8. Given the degraded nature of FFPE-derived RNA, all primers used for RT-qPCR were designed to generate short amplicons (<100 bp) to ensure efficient amplification. The treated RNA samples were then used for whole-transcriptome sequencing to investigate the genetic profiles of LG and HG patient samples relative to the control (BPH) and to each other.

### 2.3. Whole Transcriptome Sequencing of RNA Extracted from FFPE Samples

A total of 700 ng of extracted RNA was used for TurboDNase treatment to remove genomic DNA (ThermoFisher Scientific, USA). A total of 100 ng of extracted Turbo DNAase-treated RNAs were used for whole-transcriptome sequencing from 21 tissue samples (7 BPH, 7 HG, and 7 LG). Briefly, whole-transcriptome sequencing was conducted using the Ion targeted AmpliSeq Transcriptome platform, which enables targeted sequencing of approximately 21,000 human RNA transcripts. Libraries were prepared using a high-throughput multiplexed amplification strategy and sequenced on the Ion S5 XL Semiconductor Sequencer (Thermo Fisher Scientific, USA) equipped with the Ion 540 Chip (Life Technologies, Carlsbad, CA, USA) following the manufacture’s protocol. Approximately 100 ng of treated RNA underwent processing using the SuperScript VILO complementary DNA (cDNA) synthesis kit (Invitrogen Waltham, MA, USA; Cat. No. 11754050), followed by amplification of the resulting DNA using Ion AmpliSeq Transcriptome Human Gene Expression Kit (Thermo Fisher Scientific, Waltham, MA, USA; Cat. No. A26325) for library preparation. Next, following library preparation, purification was performed using Agencourt AM-Rute XP Beads (Beckman Coulter, Indianapolis, IN, USA). Library concentration was subsequently determined using the Ion Library TaqMan^TM^ Quantification Kit (Applied Biosystems, Waltham, MA, USA). The libraries were diluted to 100 pM and pooled; equal volumes of each barcoded library were combined. Template preparation and chip loading were performed on the fully automated Ion Chef System, and sequencing was carried out on the Ion S5 XL Semiconductor sequencer with the Ion 540 Chip (Thermo Fisher Scientific).

### 2.4. RNA Sequencing Data Analysis

Analysis of transcriptomic sequencing data was conducted using the Ion Torrent Software Suite version 5.4. Raw sequencing reads underwent preprocessing, including adapter trimming and removal of low-quality reads, using the integrated Ion Torrent pipeline. Processed reads were aligned to the human reference genome (hg19/GRCh37) using the Torrent Mapping Alignment Program (TMAP), which is optimized for Ion Torrent sequencing data. To enhance high alignment sensitivity and specificity, two-stage mapping strategy was applied. Initially, short reads were aligned using the Burrows–Wheeler Aligner (BWA-short), followed by refinement of alignment with the Sequence Search and Alignment by Hashing Algorithm (SSAHA). Additional polishing steps were performed using BWA-long and Smith–Waterman algorithms to improve mapping precision, specifically within complex genomic regions. This combined strategy improved alignment confidence and minimized platform-specific mapping errors. Differential expression analysis was performed using the DESeq2 package (version 1.46.0) to identify differentially expressed genes (DEGs) and all genes used for downstream analyses, including gene set enrichment analysis (GSEA) and leading-edge gene identification, were derived exclusively from DESeq2 results. Raw read counts obtained from RNA sequencing were used as input for the analysis, with normalization performed using the size factor-based method implemented in DESeq2. Genes with low expression (counts < 10) were excluded to reduce background noise. For data assessment and visualization, principal component analysis (PCA) was used, which was performed in R (version 4.4.3) using the PCAtools package (version 2.18.0). Given the exploratory nature of the study, DEGs were initially identified using a nominal significance threshold of *p*  <  0.05 and considered for exploratory prioritization. This approach was used to reduce the likelihood of excluding potentially biologically relevant genes that may not meet strict FDR thresholds. Additionally, an unpaired t-test accounting for unequal variances (*p* < 0.05) was used as a supplementary sensitivity analysis to evaluate the consistency of selected gene expression patterns under an alternative statistical framework, thereby providing an additional layer of stringency to candidate gene selection and prioritizing sensitivity in detecting probable biological signals. Furthermore, to enhance the robustness of the findings, experimental validation and gene set enrichment analysis (GSEA) was conducted to refine DEG selection based on pathway-level significance while incorporating false discovery rate (FDR) correction, thereby providing additional biological validation. Shared DEGs among different comparisons were identified using the InteractiVenn web-based tool (https://bio.tools/interactivenn) accessed on 3 November 2025.

### 2.5. Functional Enrichment Analysis by Metascape

Pathway analysis and functional clustering of the common DEGs and/or frequently occurring genes were conducted using the Metascape tool (http://metascape.org, accessed on 5 November 2025). Metascape enabled validation of pathways identified by GSEA and frequently occurring genes. This software provides comprehensive pathway enrichment and functional categorization.

### 2.6. Gene Set Enrichment Analysis

To explore pathway-level alterations associated with LG and HG disease, significant DEGs were further analyzed using absolute gene set enrichment analysis (absGSEA). The analysis was performed on the expression data in R using a previously described custom absGSEA script [25]. Gene set annotations were obtained from the Molecular Signatures Database (MSigDB; https://www.gsea-msigdb.org, accessed on 1 November 2025), which provides a comprehensive collection of curated and computationally derived pathways (approximately 90,000) from the Broad Institute’s database supplemented with additional custom gene sets. Both standard GSEA and absGSEA were performed using significance thresholds of nominal *p*-value < 0.05 and false discovery rate (FDR) *q*-value < 0.25. This step served as a secondary, more stringent pathway-level filtering approach following initial gene-level screening. The enriched gene sets were further refined through systematic cross-referencing, whereby significantly enriched genes were mapped to their corresponding pathways. Finally, genes recurrently represented across multiple significantly enriched pathways were subsequently prioritized for downstream analysis.

### 2.7. Validation of Putative Biomarkers in Independent Prostate Adenocarcinoma Patients via GEPIA2 and UALCAN

The putative biomarkers derived from RNA-seq were validated using independent cohorts from TCGA datasets consisting of a sample size of 496 through two different analyses, GEPIA2 (http://gepia2.cancer-pku.cn/#survival, accessed on 7 November 2025) and UALCAN (https://ualcan.path.uab.edu/analysis.html, accessed on 7 November 2025). GEPIA2 provides interactive analysis and visualization of gene expression, survival, and correlation data derived from RNA-seq data in the TCGA. UALCAN explores cancer omics data and enables gene expression analysis, along with clinical parameters such as tumor stage, grade, and patient survival, using the TCGA dataset.

### 2.8. Quantitiave Real-Time Polymerase Reaction (qRT-PCR)

Candidate biomarkers were further validated using qRT-PCR in fifteen independent PC cases (five from each group) along with six overlapping samples (two from each group). Complementary DNA (cDNA) was synthesized from total RNA from prostate tissues (*n* = 21), including BPH (*n* = 7), LG (*n* = 7) and HG (*n* = 7) cases, using the High-Capacity cDNA Reverse Transcription Kit (Applied Biosystems, Foster City, CA, USA) as per the manufacturers protocol. qRT-PCR was performed using a Maxima SYBR Green/ROX qPCR Master Mix (Thermo Scientific, USA) in QuantStudio3 Real-Time PCR thermal cycler (Applied Biosystems, Waltham, MA, USA). Samples were repeated in three independent technical replicas. Relative gene expression levels were calculated using the 2^−(ΔΔCt)^ method with normalization to internal housekeeping gene glyceraldehyde 3-phosphate dehydrogenase (GAPDH). Primer sets used are listed in Table 1.

### 2.9. Exploration of Immune Cell Characteristics

Immune cell signature profiling of the samples was performed using Cell-Type Identification by Estimating Relative Subsets of RNA Transcripts (CIBERSORTx) (https://cibersort.stanford.edu, accessed on 3 December 2025) [26]. This algorithm relies on predefined gene expression signatures comprising approximately 500 genes. The LM22 reference signature matrix was applied, which includes 547 genes and enables discrimination of 22 human hematopoietic cell phenotypes, including naïve and memory B cells, multiple T cell types (*n* = 7), plasma cells, natural killer cells, and various myeloid lineages. Normalized gene expression profiles from the in-situ cohorts were uploaded to the CIBERSORTx web portal for deconvolution analysis.

### 2.10. Human Protein Atlas Immunohistochemistry Data

Representative immunohistochemical staining images for the selected candidate proteins were retrieved from the pathology atlas of the Human Protein Atlas “(HPA; https://www.proteinatlas.org/ (accessed on 3 March 2026))” [27,28], an antibody-based proteomic resource that provides protein expression profiles across normal and cancer tissues. Images corresponding to prostate adenocarcinoma and normal prostate tissues were selected to illustrate the protein expression patterns of the identified biomarkers. These images were included as supportive visual evidence to complement the transcriptomic and experimental findings.

### 2.11. Statistical Analysis

Student’s t-test was conducted to determine significant differences in gene expression amongst control, LG, and HG. Additionally, to compare gene expression across subsets, Pearson’s Chi-squared test (χ2) and Fisher’s exact test (two-tailed) were used in the software tool; *p* < 0.05 was considered statistically significant. For qRT-PCR analysis, statistical comparisons between groups were performed using the Mann–Whitney test in GraphPad Prism 8.4. Data are presented as mean ± standard deviation (SD).

## 3. Results

### 3.1. Performance and Quality Metrics of Sequencing

Targeted amplicon sequencing of the PC cohort demonstrated consistently high sequencing performance and data quality across all groups. The mean number of mapped reads was 5.47 × 10^6^ in HG, 6.91 × 10^6^ in LG, and 4.52 × 10^6^ in the control samples, with comparable mean read lengths ranging from 81 to 86 bp. High-quality reads (≥Q20) were abundantly represented across all groups, indicating reliable base-calling accuracy. Overall, sequencing achieved a mean raw accuracy of 97.85%, with a substantial average coverage depth of 85.5X and 93.4% of amplicons successfully aligned to the hg19 reference genome. Based on these quality metrics, downstream analyses were performed to characterize grade-specific transcriptional alterations (Table 2).

### 3.2. Differential Gene Expression Signatures Observed Across Control, Low-Grade, and High-Grade Prostate Cancer

To characterize transcriptional alternations associated with PC development and progression, differential gene expression analysis was conducted using DESeq2 across three groups: HG, LG, and the control. As an initial step, HG and LG tumors were each compared independently with the control to identify tumor-associated transcriptional changes and potential genes associated with disease development.

Compared with the control group, a distinct gene expression profile was identified, with 3518 significantly differentially expressed genes (DEGs) in the HG and 1798 DEGs in the LG (Table S2A and S2B, respectively). Differential expressions were defined as *p* < 0.05 and log2FC > 1 and log2FC < −1. The volcano plots illustrating the distribution of upregulated and downregulated genes are shown in Figure S1A,B.

To assess global expression profiles, unsupervised hierarchical clustering was performed using the identified DEGs from each comparison. Heatmaps revealed a clear separation between tumor and control samples, with the distinct clustering of genes in both the LG—control (Figure S1C) and HG–control (Figure S1D) comparisons, reflecting distinct tumor-associated transcriptional profiles within each grade. These analyses establish a robust tumor-specific expression profile, which is further supported by the identification of shared and grade-dependent transcriptional alterations.

### 3.3. Shared Oncogenic and Inflammatory Transcriptional Features Across Tumor Grades

**Figure 2:**
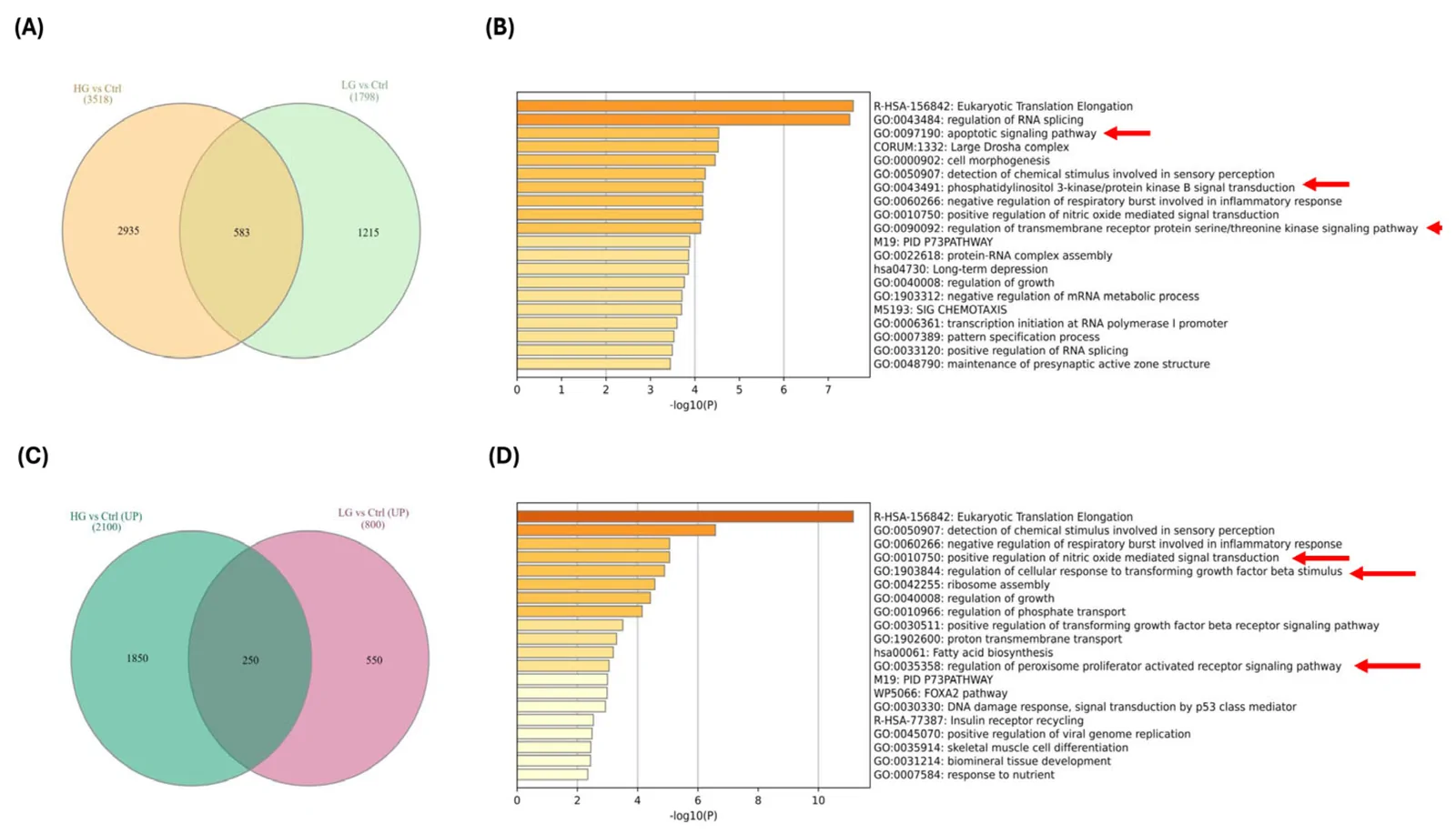
Shared DEGs, upregulated genes, and enriched pathways among LG vs. control and HG vs. control tumor comparisons. (**A**) Venn diagram illustrating the overlap of DEGs among both comparisons, revealing 583 common DEGs. (**B**) Bar plot showing the top significantly enriched pathways using the Metascape functional analysis based on the shared DEGs identified. Enrichment of pathways associated with RNA processing and transcriptional regulation (highlighted by red arrows). (**C**) A Venn diagram illustrates the overlap of the statistically significant upregulated genes among both comparisons, revealing 250 shared genes. (**D**) Enriched biological pathways using Metascape were identified based on the shared upregulated genes, revealing enrichment of stress response and growth factor-responsive pathways (highlighted by red arrows).

To identify transcriptional features that emerge early during tumorigenesis and are maintained across disease grades, DEGs from the HG–control and LG–control comparisons were compared to identify overlapping genes. This analysis revealed 583 shared DEGs between the two comparisons, representing genes dysregulated in tumors (Figure 2A, Table S3A).

Functional enrichment analysis of the shared DEGs obtained from HG and LG comparisons with the control revealed significant enrichment for pathways associated with tumorigenesis, including the apoptotic signaling pathway, the phosphatidylinositol 3-kinase/protein kinase B signal transduction, and the regulation of transmembrane receptor protein serine/threonine kinase signaling pathway (Figure 2B, red arrows). Collectively, these findings highlight early and sustained transcriptional alterations associated with the activation of oncogenic and inflammatory signaling pathways.

To refine these alterations and focus on genes most likely to contribute to tumorigenesis, our analysis next identified genes that were consistently upregulated across both comparisons. This analysis revealed 250 shared upregulated genes in both LG and HG tumors when compared to the control (Figure 2C, Table S3B), defining a transcriptional signature common across tumor grades. These genes were found to be associated with the enrichment of growth factor-responsive pathways and immune-stress regulatory processes, including the positive regulation of nitric oxide-mediated signal transduction, regulation of cellular responses to the transforming growth factor beta stimulus, positive regulation of the transforming growth factor beta receptor signaling pathway, and regulation of the peroxisome proliferator-activated receptor signaling pathway (Figure 2D, red arrows). These findings suggest that, beyond the broad transcriptional dysregulation, a distinct immune-modulatory and stress-adaptive program is established early and maintained throughout disease progression.

### 3.4. IL2RA and SLC29A2 Are Clinical Candidates Associated with Prostate Progression

Given the enrichment of immune-related processes, shared upregulated genes were further compared with the gene frequency list derived from C7 immunological signatures in both HG–control and LG–control analyses. This comparison identified five consistently significant upregulated genes, including *HSPA1A, SLC29A2, NUDT18, B3GAT1*, and *IL2RA* (Table S4). These genes include *HSPA1A* [29], *SLC29A2* [30], *NUDT18* [31], *B3GAT1* [32], and *IL2RA* [33], which are functionally associated with cellular stress, metabolic regulation, and immune regulation.

To assess the clinical relevance of these shared candidate genes, the expression of *HSPA1A, SLC29A2, NUDT18, B3GAT1*, and *IL2RA* genes were evaluated in an independent PRAD cohort from TCGA and GTEx using ULCAN and GEPIA2 platforms. Gene expression levels were examined across different Gleason scores and evaluated for associations with overall survival (OS) and disease-free survival (DFS).

**Figure 3:**
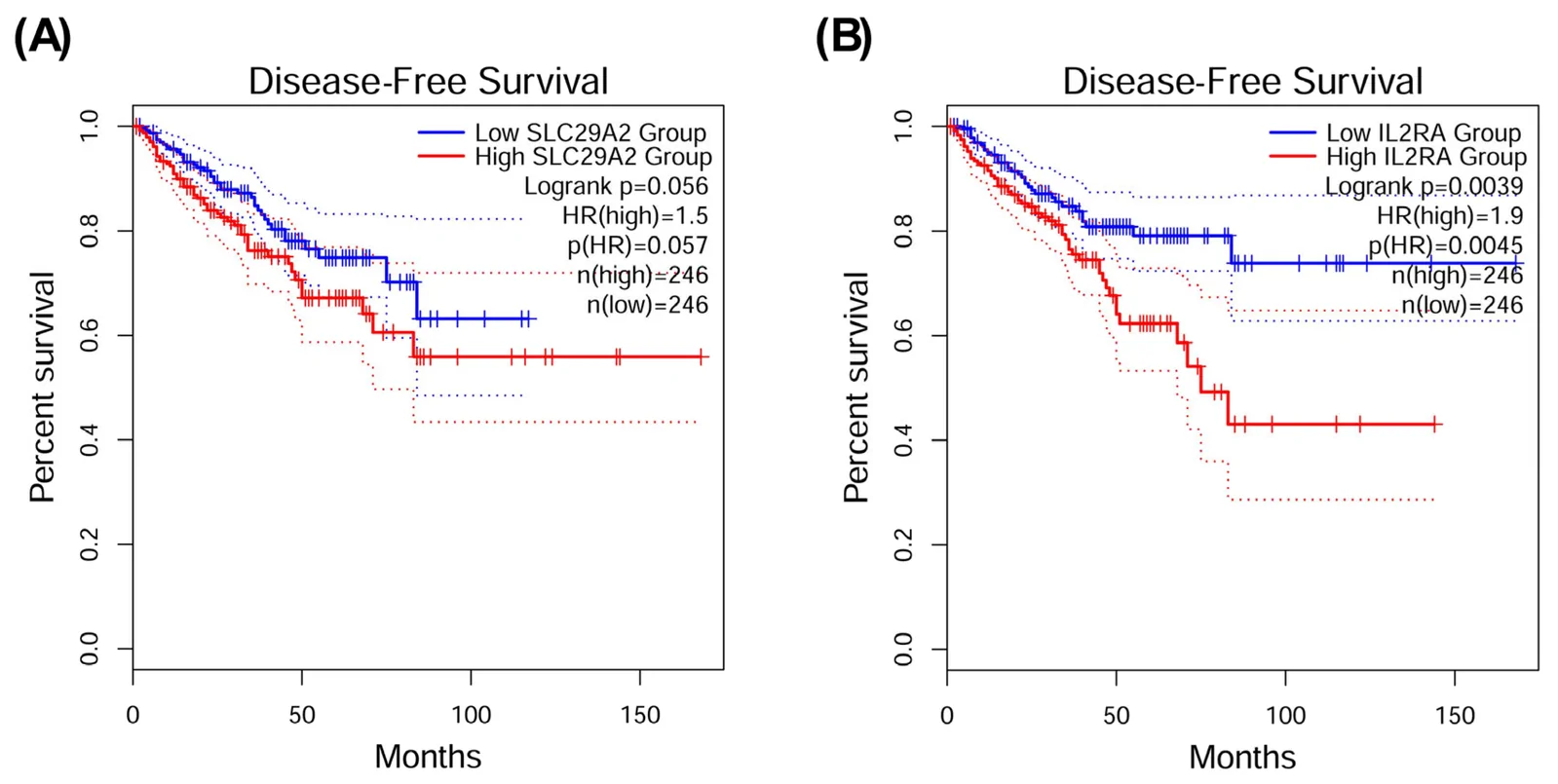
Clinical relevance of SLC29A2 and IL-2RA as candidate genes. Disease-free survival analysis within the PRAD cohort using GEPIA2 revealed poor DFS was associated with higher expression of (**A**) SLC29A2 and (**B**) IL2RA. The cut-off of the higher and lower expression cohorts was the median, and the red and blue lines represent the higher and lower expression of respective genes, respectively.

**Figure 4:**
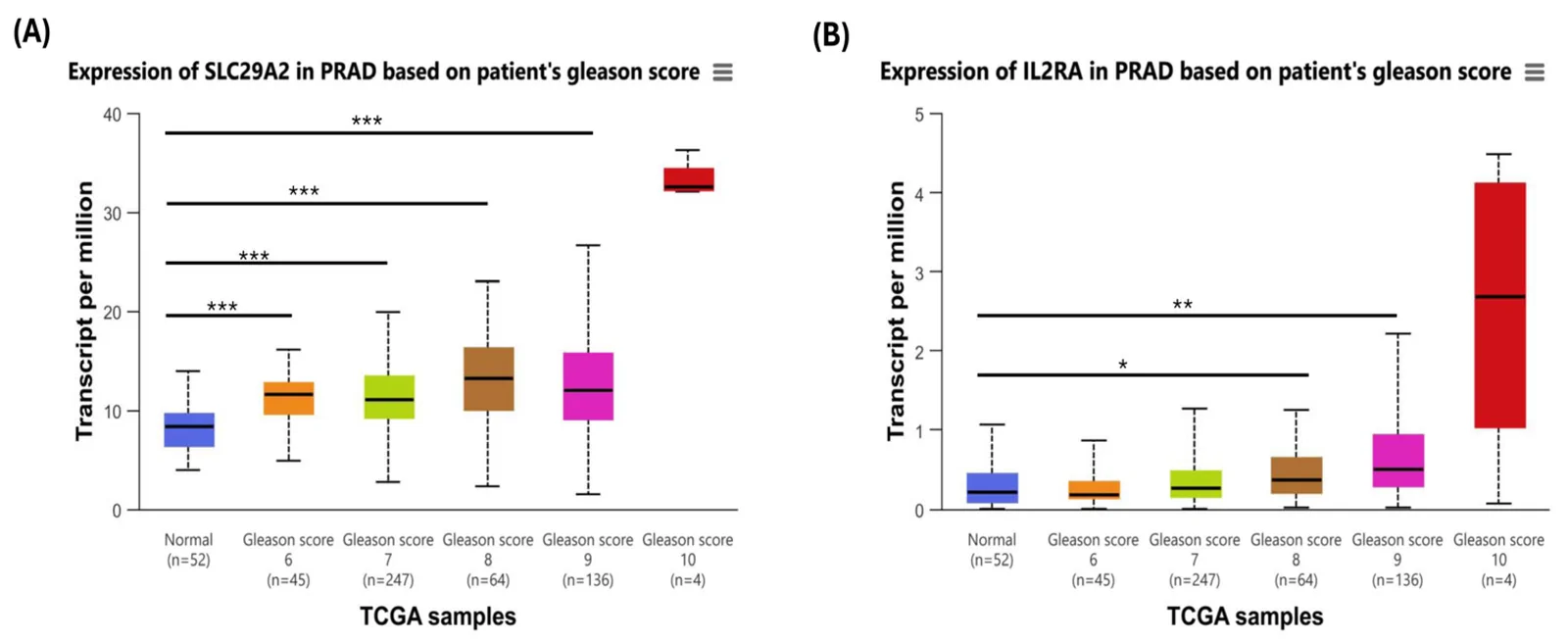
Clinical relevance of SLC29A2 and IL-2RA as candidate genes. Confirmation of the gene expression across Gleason grades compared to normal in the PRAD cohort using the UALCAN platform. Both genes, (**A**) SLC29A2 and (**B**) IL2RA, showed a markedly significant increase in expression during disease progression. Figures were generated using the UALCAN database. * *p* < 0.05, ** *p* < 0.001, and *** *p* < 0.0001 were considered statistically significant. Colors indicate patient groups, including normal and Gleason score categories (6–10).

The analysis revealed that higher expression levels of *IL2RA* and *SLC29A2* were associated with a shorter DFS (Hazard Ratio >1, *p (HR) ≤ 0.05*, *n* = 492) (Figure 3A,B). Notably, *SLC29A2* expression showed a significant association with reduced OS, whereas *IL2RA* did not demonstrate a significant correlation with OS (Figure S2A,B). Both genes exhibited a progressive increase in expression with higher Gleason scores, indicating a positive association with tumor progression (Figure 4A,B and Figure S3A–D). While *IL2RA* has been minimally studied in PC, and it is linked to immune-related mechanisms [34,35], the role of *SLC29A2* in PC remains largely unexplored. Therefore, *SLC29A2* is proposed here as a putative candidate biomarker associated with PC progression, warranting further functional and mechanistic validation.

### 3.5. Global Transcriptomic Profiling Reveals a Pronounced Grade-Dependent Molecular Signature

Given the transcriptional alterations common to both LG and HG tumors relative to the controls, the next focus was to identify grade-specific molecular alterations associated with disease progression by comparing LG and HG tumors using whole-transcriptome RNA-seq analysis. This comparison was performed independently of the control samples. Principal component analysis (PCA) clearly separated LG and HG tumors, supporting the presence of distinct grade-associated transcriptional profiles (Figure S4A). Differential expression analysis using DESeq2 identified 2296 significant DEGs (Table S5) (*p* < 0.05; log2FC > 1 and log2FC < −1), including 1304 upregulated and 923 downregulated genes in HG compared to LG tumors (Figure S4B). Unsupervised hierarchical clustering of these DEGs revealed two distinct expression clusters corresponding to tumor grade, as indicated by black boxes (Figure S4C). Cluster 1 comprises genes highly expressed in LG tumors compared to HG tumors, while cluster 2 represents genes with higher expression in HG tumors compared to LG tumors. These findings highlight a grade-associated molecular signature that directs downstream functional analysis.

Expression patterns of well-established PC markers validated the biological relevance of our dataset. *AMACR* and *NEK2* are associated with aggressive disease and positive correlation with Gleason progression and have been found to be significantly upregulated in HG tumors [36,37,38]. Conversely, *PRDM2*, which encodes the tumor-suppressive RIZ1 isoform [39] and has been reported to negatively correlate with the increasing Gleason score, was significantly downregulated in HG tumors (Table S6).

### 3.6. Functional Enrichment Analysis Reveals a Grade-Associated Phenotypic Reprogramming

**Figure 5:**
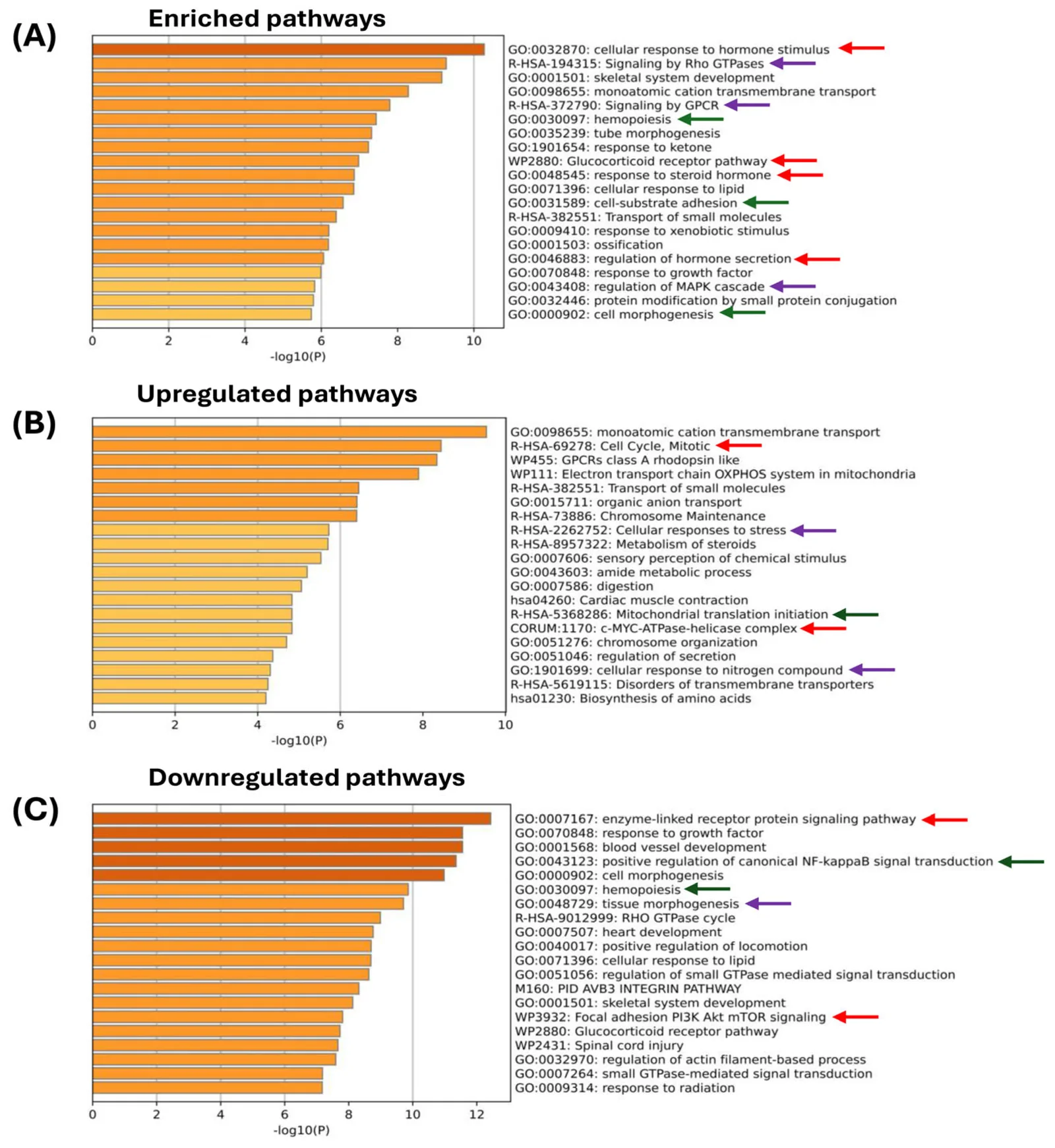
Functional enrichment analysis across disease grades. Bar plots showing the top significantly enriched biological processes and signaling pathways identified by Metascape. (**A**) Functional enrichment analysis of the differentially expressed genes (DEGs) reveals enrichment of pathways related to hormone response, developmental processes, and cellular morphogenesis. (**B**) Metascape enrichment analysis of genes upregulated in HG tumors highlights pathways associated with cell-cycle regulation, mitochondrial function, metabolic processes, and transmembrane transport. (**C**) Metascape enrichment analysis of genes downregulated in HG tumors shows relative enrichment of receptor-mediated signaling, immune-associated pathways, including canonical NF-κB signaling, and developmental programs that are more prominent in LG tumors. Bar plots are ranked by statistical significance (−log10 *p*-value). The color gradient reflects increasing statistical significance, with darker shades indicating stronger enrichment. Colored arrows indicate representative pathways.

Functional enrichment analysis of DEGs from HG versus LG using Metascape revealed the broad dysregulation of hormone-responsive pathways, including cellular response to the hormone stimulus, glucocorticoid receptor pathway, response to the steroid hormone, and regulation of hormone secretion (Figure 5A, red arrows).

The upregulated genes in HG tumors (Table S7A) showed enriched pathways related to cell-cycle progression, including cell cycle, mitotic, and the c-MYC-ATPase-helicase complex (Figure 5B, red arrows). In addition, pathways related to mitochondrial function were enriched, including the electron transport chain OXPHOS system in mitochondria and mitochondrial translation initiation (Figure 5B, green arrows). Upregulation of stress response pathways, including cellular responses to stress and cellular responses to the nitrogen compound, were observed (Figure 5B, purple arrows). These findings are consistent with the transcriptional patterns associated with high proliferative and metabolic activity in aggressive disease [40].

In contrast, genes downregulated in HG tumors (Table S7B) were enriched in receptor-mediated signaling pathways such as the enzyme-linked receptor protein signaling pathway (Figure 5C, red arrow), positive regulation of canonical NF-kappaB signal transduction (Figure 5C, green arrow), and focal adhesion PI3K -Akt- mTOR signaling (Figure 5C, purple arrow). Collectively, these patterns suggest a grade-associated transcriptional shift, with reduced representation of immune-related signaling and increased representation of proliferative programs.

To further explore pathway dynamics across disease grades, absolute GSEA was performed on the DEGs identified from HG compared to LG comparison across MSigDB collections (C1–C8). Using a nominal *p-value* threshold of <0.05 and biological relevance, significant enrichment was observed across multiple collections. Specifically, 54 pathways were identified in C2, 140 pathways in C5, and 287 pathways in C7 (Table S8).

**Figure 6:**
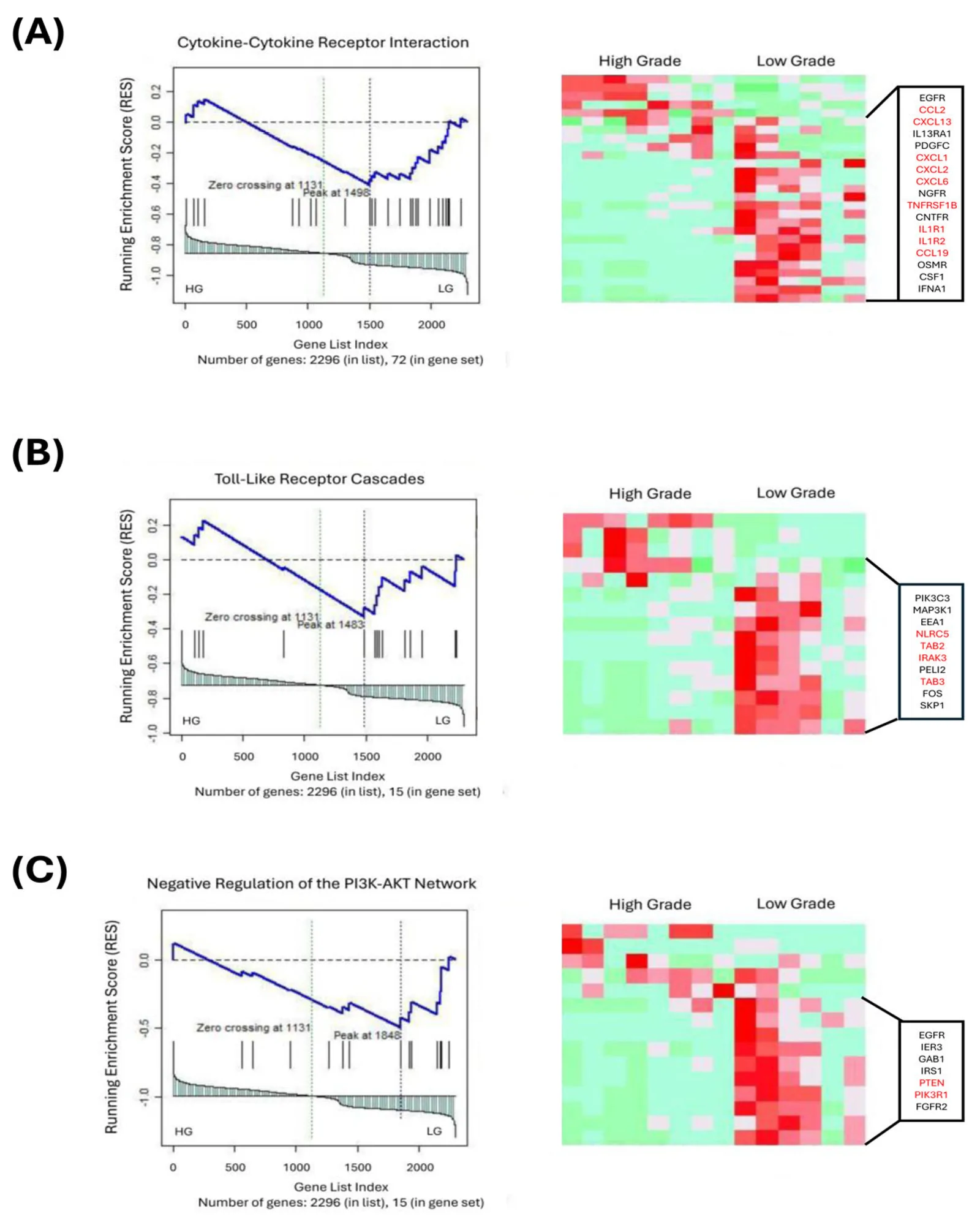
Selected pathways identified by GSEA from HG vs. LG tumor comparison. Enriched pathways from the C2 collection, including (**A**) cytokine–cytokine receptor interaction, (**B**) Toll-like receptor (TLR) cascades, and (**C**) negative regulation of PI3K/AKT signaling. Heatmaps demonstrate the expression profiles of genes within representative pathways. Red color reflects high gene expression levels, while green color reflects low gene expression. Highlighted genes within the boxes were selected for further study based on their higher frequency across enriched pathways and their functional relevance.

Within the C2 gene sets, LG tumors showed the marked enrichment of cytokine–cytokine receptor interactions with key immune mediators, including *TNFRSF1B (TNFR2), IL1R1, IL1R2, CCL2, CCL19*, and several CXCL chemokines (*CXCL1, CXCL2, CXCL6,* and *CXCL13*), showed lower expression in HG tumors (Figure 6A). Enrichment of Toll-like receptor (TLR) cascades in LG tumors includes the TLR-associated adaptor and regulatory genes such as *TAB2, TAB3* [41], *IRAK3* [42], and *NLRC5* [43,44], suggesting the differential representation of innate immune-related transcriptional programs between grades (Figure 6B).

In parallel, the negative regulation of PI3K/AKT signaling was enriched in LG tumors, characterized by the higher expression of tumor suppressors and PI3K/AKT negative regulators, including *PTEN* [45] and *PIK3R1* [46], in LG tumors compared to HG tumors (Figure 6C). To prioritize candidate genes associated with PI3K/AKT pathway dysregulation in PC, genes within the C2 gene frequency table were screened based on three criteria: selective upregulation in HG compared with LG tumors, reported functional involvement in PI3K/AKT signaling in other cancer types, and limited prior characterization in PC. Based on these criteria, *AAMDC* and *ASAH2* were selected as candidate genes potentially associated with PI3K/AKT-related transcriptional alterations in HG tumors (Table S9).

### 3.7. GSEA Revealed Grade-Associated Enrichment in NF-κB Pathway Activity

**Figure 7:**
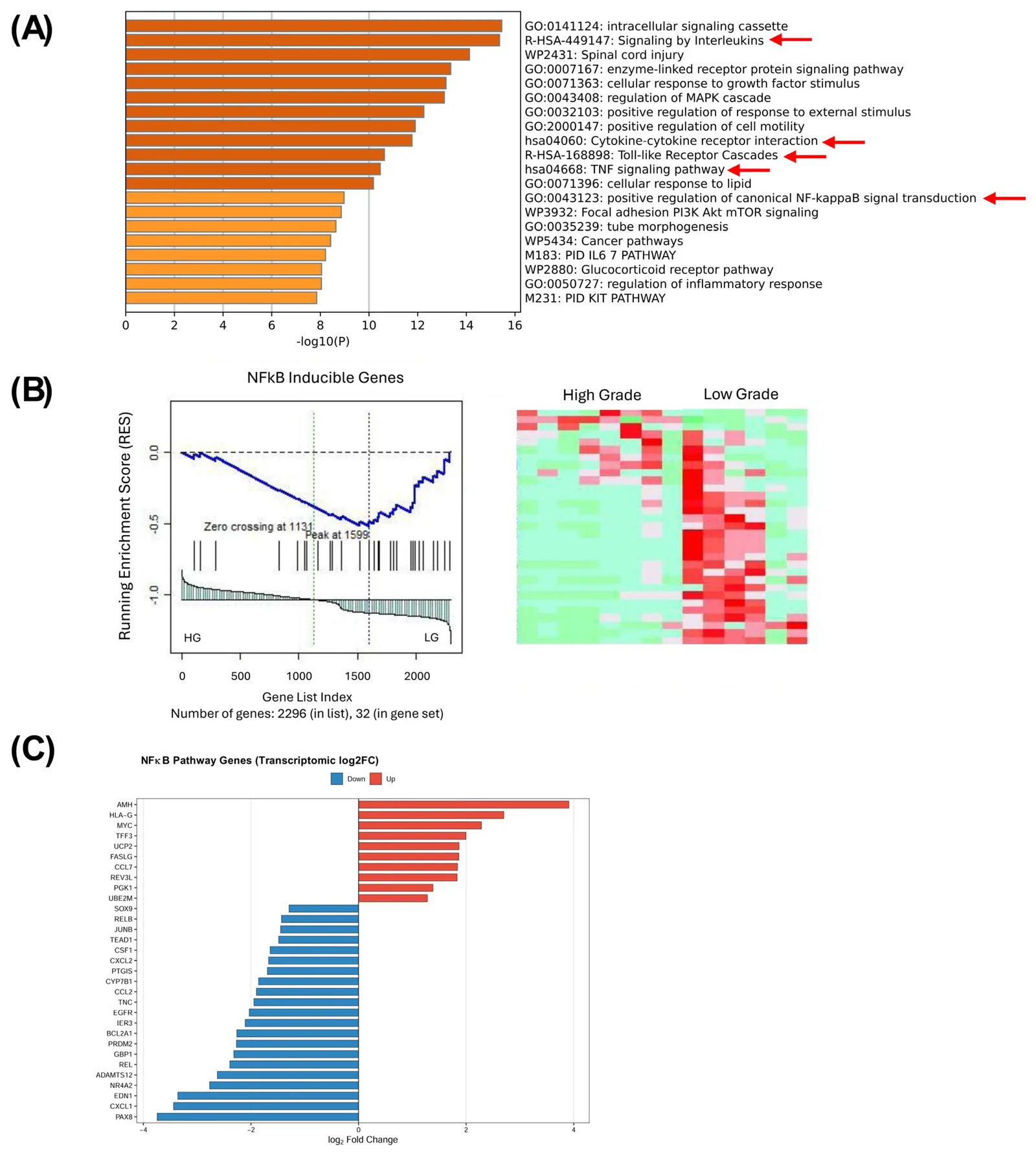
NF-κB-associated pathway enrichment and gene expression patterns in HG vs. LG tumor comparison. (**A**) Metascape functional enrichment analysis based on gene frequency within the C2 gene set collection, highlighting prominent enrichment of interleukin signaling, cytokine–cytokine receptor interaction, Toll-like receptor (TLR) cascades, TNF signaling, and positive regulation of canonical NF-κB pathways (highlighted by red arrows). (**B**) GSEA heatmap of NF-κB-inducible gene sets, demonstrating significant negative enrichment in HG tumors. Heatmaps show expression profiles of genes within representative pathways. Red indicates high expression, while green indicates low expression. (**C**) Log2 fold-change (log2FC) of significantly up- and downregulated NF-κB-inducible genes in the HG vs. LG tumor comparison.

To further characterize grade-associated biological processes, gene frequency analysis was conducted to identify key drivers within enriched pathways. Enrichment analysis of high-frequency genes identified from the gene frequency analysis of the C2 gene set revealed the significant enrichment of pathways related to interleukin signaling, cytokine–cytokine receptor interaction, TLR cascades, TNF signaling, and the positive regulation of canonical NF-κB pathways (Figure 7A, red arrows). These findings were consistent with GSEA results and highlight the prominent representation of NF-κB-associated transcriptional programs across tumor grades.

Consistent with this observation, GSEA using the NF-κB-inducible gene set showed significant negative enrichment in HG tumors (Figure 7B). Gene-level analyses further revealed the broad downregulation of inflammatory NF-κB target genes in HG tumors, with selected stress-adaptive or immune-modulatory NF-κB targets remaining expressed (Figure 7C). Together, these findings suggest the differential representation of NF-κB-related transcriptional programs in HG tumors, with a relative shift from inflammatory immune-associated gene expression toward the enrichment of gene signatures commonly associated with tumor survival.

To gain insight into the regulatory basis underlying the transcriptional patterns associated with attenuated inflammatory NF-κB signaling in HG tumors, we next examined changes in the expression of key upstream receptors and NF-κB regulatory components in the DEGs dataset. NF-κB-associated genes were defined using a curated list of NF-κB target genes maintained by the Gilmore laboratory at Boston University [47].

Several regulatory alterations were identified, including differential expressions of the upstream TNF receptor *TNFRSF1B* and NF-κB regulatory components *NFKBIL1* and *NFKBIZ* (Table S10). *TNFRSF1B*, a stabilizer of NF-κB-inducing kinase (NIK) and activator of non-canonical NF-κB signaling [48], was significantly downregulated in HG tumors. In contrast, *NFKBIL1*, an atypical IκB-like regulator of TLR/IRF-mediated NF-κB transcription [49], was significantly upregulated in HG tumors. Moreover, *NFKBIZ*, a nuclear NF-κB co-activator [50], was significantly downregulated in HG tumors. Collectively, these findings are consistent with grade-associated differences in NF-κB-related transcriptional profiles, including attenuated inflammatory NF-κB outputs in HG tumors.

### 3.8. Gene Frequency Analysis Revealed Proliferative Signatures in HG Tumors

GSEA using the C5 gene ontology collection demonstrated prominent pathway enrichment in LG tumors, including the negative regulation of programmed cell death and regulation of growth (Figure S5A,B). To identify genes associated with HG phenotypes, gene frequency analysis was performed on genes represented in C5-enriched pathways, focusing on those present in more than five distinct pathways enriched for proliferation and survival.

**Figure 8:**
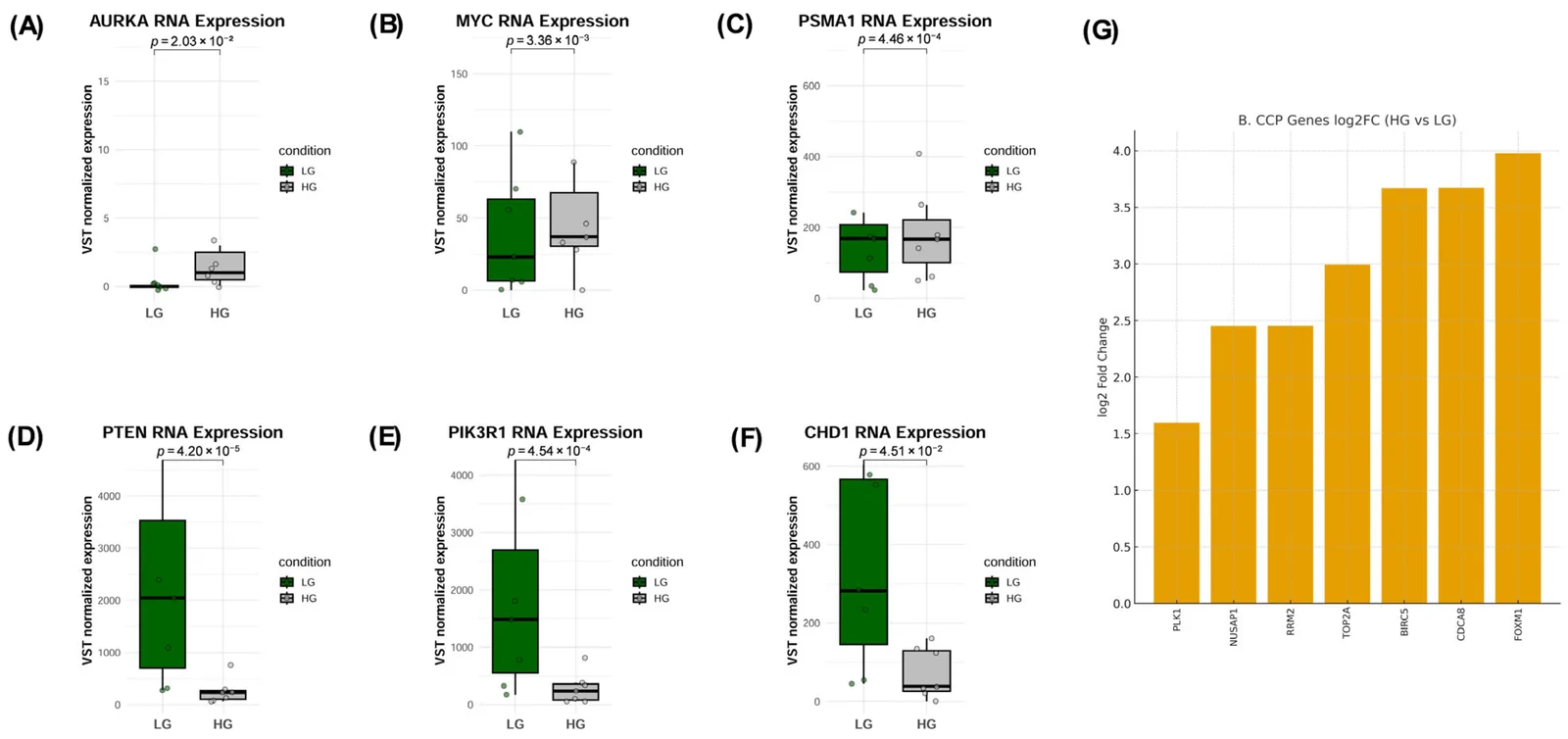
Genes associated with HG tumor phenotypes. Gene frequency analysis of C5-enriched pathways was used to identify genes recurrently involved in proliferation and survival-related processes. Boxplots show the expression levels of proliferation and survival-associated genes (**A**) AURKA, (**B**) MYC, and (**C**) PSMA1, together with a tumor suppressor gene panel comprising (**D**) PTEN, (**E**) PIK3R1, and (**F**) CHD1. These boxplots illustrate statistically significant differences in gene expression. (**G**) log_2_FC of Prolaris^®^ cell-cycle progression genes comparing HG and LG tumors.

This analysis identified several genes associated with proliferative and survival-related processes that were significantly upregulated in HG tumors, including *AURKA*, *MYC*, and *PSMA1*. In parallel, several tumor suppressor genes were downregulated, including *PTEN*, *PIK3R1*, and *CHD1* (Figure 8A–F). Although *PSMA1* has been associated with tumor cell survival in other cancers, its contribution to prostate cancer biology remains largely unexplored [51]. Consistent with these findings, Prolaris^®^ cell-cycle progression genes [52] were markedly upregulated in HG tumors, including *TOP2A, BIRC5, CDCA8, FOXM1, PLK1, RRM2,* and *NUSAP1* (Figure 8G). These results indicate that the gene-level analysis suggests the presence of a proliferative transcriptional signature within HG tumors.

### 3.9. Experimental Validation of the Candidate Biomarkers Uusing qRT-PCR in FFPE Prostate Specimens

**Figure 9:**
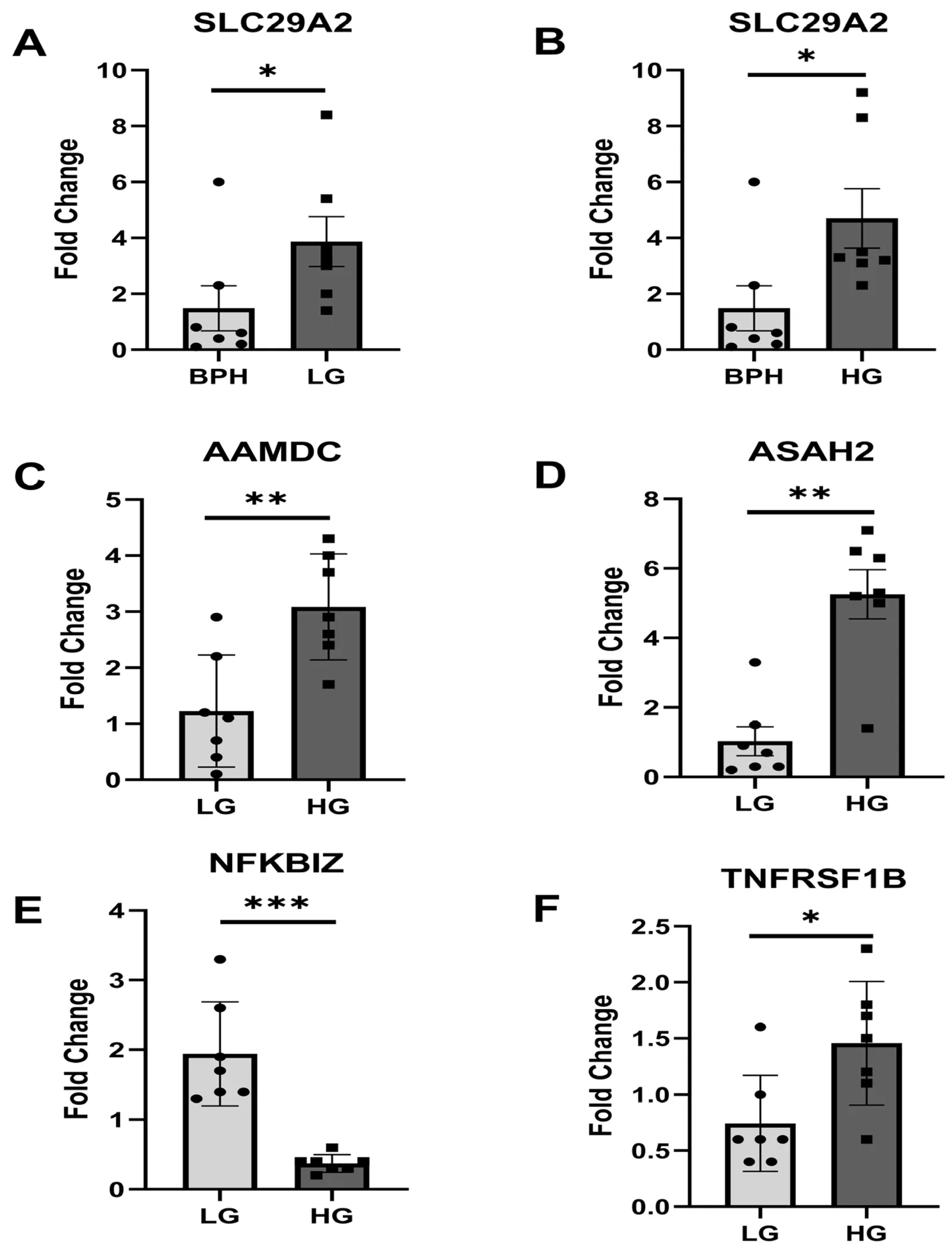
qRT-PCR validation of candidate genes identified by RNA-seq in the FFPE prostate specimens. Bar graphs showing fold-change expression of five candidate genes in FFPE prostate tissues from BPH, LG, and HG patients, validated using qRT-PCR. (**A**,**B**) SLC29A2: Significantly elevated expression in both LG and HG compared with BPH (*p* = 0.0379 and *p* = 0.0122, respectively). (**C**,**D**) AAMDC and ASAH2: Significantly higher expression in HG compared with LG (*p* = 0.0076 and *p* < 0.0023, respectively). (**E**,**F**) TNFRSF1B and NFKBIZ: Significantly reduced expression in HG compared with LG (*p* = 0.0192 and *p* = 0.0006, respectively). Statistical analysis was performed using the Mann–Whitney test. A *p*-value ≤ 0.05 was considered statistically significant. Data are presented as mean ± SD. * *p* < 0.05; ** *p* < 0.01; and; *** *p* < 0.001.

To validate the transcriptomic findings, qRT-PCR was conducted on FFPE prostate tissues from BPH, LG, and HG patients. Candidate genes were initially prioritized based on consistent differential expression across comparisons and their relevance to key transcriptional patterns associated with the tumor grade, aligning with the study objective of identifying grade-associated changes. An independent (unpaired) t-test was subsequently applied as a complementary sensitivity analysis to assess the consistency of expression patterns among the shortlisted genes. Based on this combined assessment, five genes (SLC29A2, AAMDC, ASAH2, TNFRSF1B, and NFKBIZ) were selected for qRT-PCR validation. qRT-PCR analysis confirmed statistically significant differences in expression for all five genes across the respective comparisons (Figure 9). *SLC29A2* demonstrated a significantly elevated expression in both LG and HG tumors relative to BPH (*p* = 0.0379 and *p* = 0.0122, respectively; Figure 9A,B). *AAMDC* and *ASAH2* were significantly upregulated in HG tumors compared with LG tumors (*p* = 0.0076 and *p* = 0.0023, respectively; Figure 9C,D). In contrast, *TNFRSF1B* and *NFKBIZ* showed significantly reduced expression in HG tumors compared with LG tumors (*p* = 0.0192 and *p* = 0.0006, respectively; Figure 9E,F). Overall, the qRT-PCR results were consistent with transcriptomic data, highlighting the reproducibility of these expression patterns across independent FFPE prostate specimens.

### 3.10. Protein-Level Assessment of Candidate Biomarkers Using Human Protein Atlas Immunohistochemistry

To provide preliminary protein-level context for the transcriptomic findings, representative immunohistochemical images were retrieved from the Human Protein Atlas (HPA; https://www.proteinatlas.org (accessed on 3 March 2026))”. This analysis is descriptive and intended as supportive, hypothesis-generating evidence rather than quantitative validation. Representative images for SLC29A2, AAMDC, ASAH2, TNFRSF1B, and NFKBIZ were examined to descriptively illustrate protein localization and relative staining patterns in normal prostate tissue and PRAD samples, including LG and HG cases.

SLC29A2 showed weak staining in normal prostate epithelium, and moderate cytoplasmic and membranous staining in both LG and HG adenocarcinoma, involving more than 75% of tumor cells (Figure S6A). AAMDC exhibited minimal staining in LG tumors, whereas HG adenocarcinoma showed moderate cytoplasmic and membranous staining involving approximately 25–75% of tumor cells (Figure S6B). ASAH2 was not detected in LG adenocarcinoma, while HG tumors demonstrated low cytoplasmic and membranous staining with weak intensity in 25–75% of tumor cells (Figure S6C).

In contrast, TNFRSF1B displayed moderate cytoplasmic and membranous staining in LG adenocarcinoma involving more than 75% of tumor cells, with reduced staining intensity in HG disease (Figure S6D). NFKBIZ staining was not detected in representative LG or HG tumor samples (Figure S6E). Collectively, these observations are descriptive and presented as representative protein-level observations.

### 3.11. Grade-Associated Immune Cell Remodeling in Prostate Cancer

**Figure 10:**
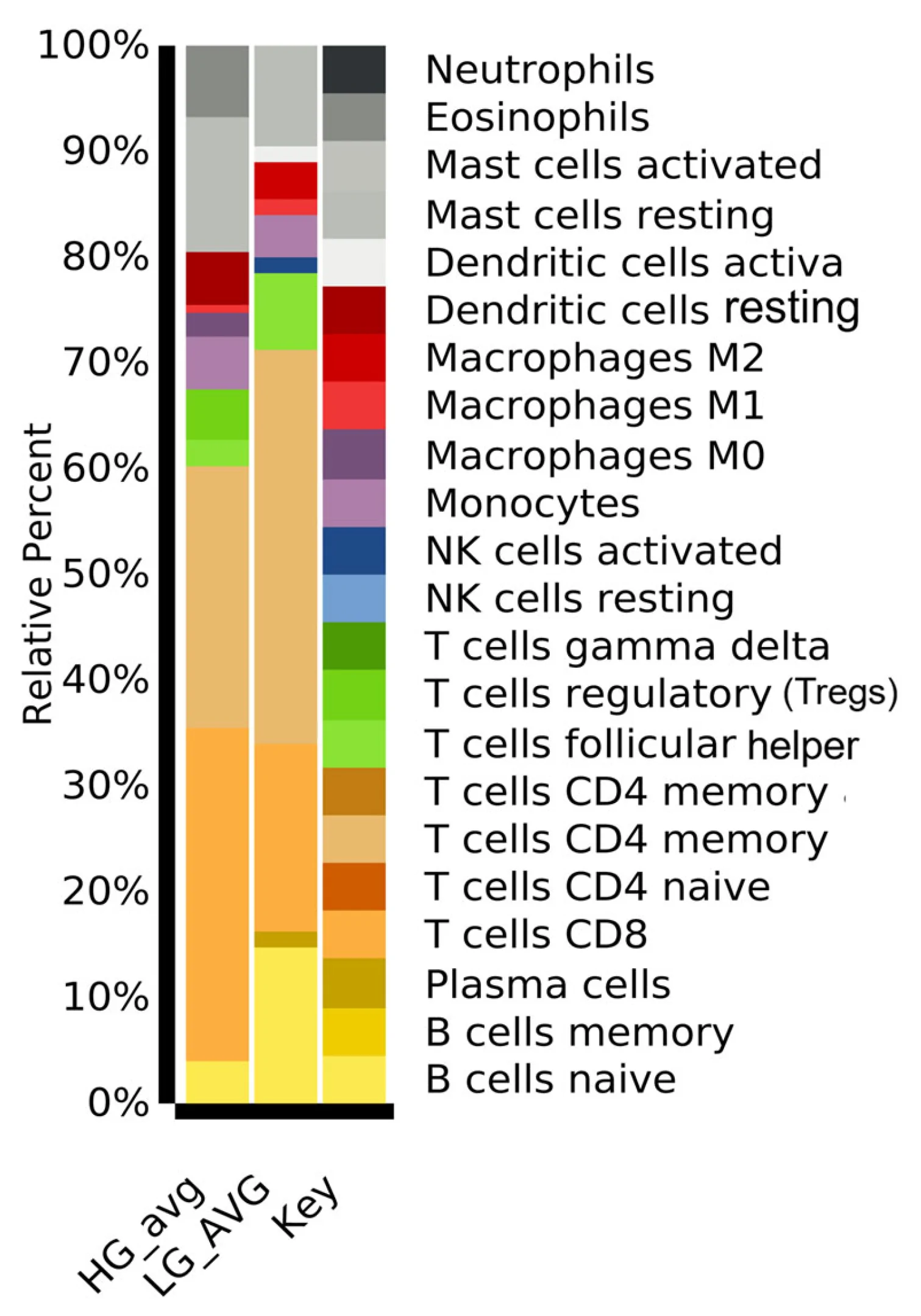
CIBERSORTx-based immune cell profiling across HG and LG tumors. Relative proportions of immune cell types using the LM22 reference signature matrix.

CIBERSORTx, a deconvolution-based computational method, was used to estimate immune cell proportions from bulk transcriptomic data. The analysis suggested distinct differences in immune cell composition between HG and LG tumors (Figure 10 and Table S11). LG tumors exhibited higher estimated proportions of adaptive immune populations, including naïve B cells (15%), resting CD4 memory T cells (37%), and follicular helper T cells (7%), consistent with increased immune-related transcriptional activity. In contrast, HG tumors were characterized by increased proportions of CD8^+^ T cells (33%), M0 macrophages (7%), and innate immune populations, including resting mast cells (18%) and eosinophils (5%), suggesting a dysregulated, coordinated immune landscape in HG tumors. These findings align with transcriptomic findings, indicating that LG tumors exhibit a broader representation of adaptive and innate immune cell subsets, whereas progression to HG disease is accompanied by immune remodeling rather than immune activation. It is important to note that these estimates should be interpreted as exploratory inferences rather than direct measurements of immune cell composition.

## 4. Discussion

PC is a clinically and molecularly heterogeneous disease, ranging from indolent tumors to aggressive forms associated with poor outcomes [3,9]. Although Gleason grading remains the most established predictor of prognosis [53], tumors with similar histopathological scores often display markedly different biological behavior, reflecting underlying molecular diversity. In this context, integrating transcriptomic profiling with pathology-based stratification offers an opportunity to better characterize the biological programs associated with grade progression. Importantly, given the use of bulk RNA sequencing, the findings presented here reflect average transcriptional signals across heterogenous tissue samples and do not resolve cell-type-specific contributions within the TME. Accordingly, rather than attempting to establish mechanistic causality, the present study aimed to define transcriptional signatures linked to histopathological progression in a pathology-annotated MENA cohort, thereby providing a molecular context for Gleason grade differences.

Using a pathology-guided design, we identified transcriptional programs that distinguish LG from HG tumors and align with known features of disease progression. Transcriptional alterations shared across tumor grades relative to benign tissues suggest early molecular changes associated with tumor development, whereas grade-specific differences point to transcriptional shifts linked to increased tumor aggressiveness. Importantly, interpreting these findings relies on pathway-level convergence and external cohort validation rather than isolated gene-level observations, supporting the biological relevance of the identified signatures while acknowledging the exploratory nature of the dataset. This framework positions transcriptomic analysis as a tool for interpreting grade-associated biology rather than as a purely discovery-driven endpoint.

Among the shared transcriptional alterations, *SLC29A2* and *IL2RA* emerged as candidate genes showing consistent upregulation across tumor grades and concordant expression patterns in independent cohorts with reproducible associations with clinical parameters. These findings support their potential relevance as grade-associated transcriptional features and align with previous evidence implicating immune and metabolic pathways in PC biology [54]. *SLC29A2*, a solute carrier family member encoding a nucleoside transporter [30], has been associated in hepatocellular carcinoma with advanced stage, invasion, and poor prognosis [55], and its preclinical targeting has improved therapeutic responses in murine models [56]. *IL2RA* (CD25), a key immune regulatory receptor subunit, exhibits increased immunoreactivity in prostate tumor tissues compared with BPH [33,57], although its grade-specific pathological relevance remains unclear. While their functional roles in PC remain incompletely defined, the consistency of their expression across datasets suggests that they may represent stable transcriptional features of disease progression rather than cohort-specific observations or definitive prognostic biomarkers, thereby warranting further functional and mechanistic investigation.

Direct comparison of HG and LG tumors further revealed transcriptional profiles consistent with increased proliferative activity and altered immune-related signaling in HG disease. Pathway-level analyses showed the enrichment of cell-cycle and metabolic programs in HG tumors, whereas inflammatory and NF-κB-associated transcriptional signatures were expressed at lower levels, reflecting differential pathway-associated transcriptional states rather than direct evidence of pathway activation or functional regulation.

Consistent with this, LG tumors demonstrated a higher expression of inhibitory regulators, including PTEN and PIK3R1, corresponding to gene sets associated with the negative regulation of PI3K/AKT signaling. In contrast, HG tumors exhibited the relative enrichment of *AAMDC* and *ASAH2*. Both transcripts have been previously associated in other malignancies with metabolic adaptation and AKT-related processes. *AAMDC* has been implicated in sustaining tumor survival under metabolic stress through the modulation of PI3K/AKT/mTOR signaling in breast cancer [58]; however, its biological relevance in PC remains largely unexplored. Similarly, *ASAH2* encodes neutral ceramidase, a regulator of sphingolipid metabolism associated with enhanced AKT signaling and reduced ceramide-mediated apoptosis in colorectal malignancies [59,60]. While PC sphingolipid research has primarily focused on acid ceramidase (*ASAH1*) [61], the relative enrichment of *ASAH2* in HG tumors suggests the potential involvement of additional lipid-metabolic regulators in advanced disease states. Within this pathology-driven transcriptomic context, the concurrent upregulation of *AAMDC* and *ASAH2* characterizes HG tumors and supports their potential relevance in grade progression, highlighting them as candidates for further biological evaluation in PC.

Another feature of grade progression is the alteration of inflammatory transcriptional programs. C2 pathway enrichment revealed the prominent enrichment of cytokine signaling, TLR cascades, TNF signaling, and canonical NF-κB pathways in LG tumors. The GSEA of the NF-κB-inducible gene set demonstrated negative enrichment in HG tumors, suggesting the reduced transcriptional representation of immune- and inflammatory-related NF-κB-associated gene signatures during disease progression rather than direct evidence of decreased pathway activity. This transition was further supported by the downregulation of *TNFRSF1B* (*TNFR2*) and *NFKBIZ* and by the upregulation of *NFKBIL1* in HG tumors. Given the established role of the *TNF-α/TNFR2* axis in immune tolerance and NF-κB pathway regulation [48], the reduced expression of *TNFRSF1B* observed in this study is consistent with previous findings. Histopathological studies have reported an inverse association between *TNFRSF1B* protein expression and proliferative activity in prostate tissue [62], with additional evidence linking reduced *TNFRSF1B* expression to increased prostate gland volume [63].

*NFKBIZ*, an unconventional IκB-like regulator that attenuates inflammatory NF-κB transcriptional programs [64,65], has been reported as a tumor suppressor in hepatocellular carcinoma, where its loss enhances tumor growth, invasion, and metastasis through NF-κB pathway regulation [50]. Based on these observations, *NFKBIZ* may have a similar functional relevance in PC, although this requires further investigation. In contrast, although *NFKBIL1* has been described as a prognostic marker in hepatocellular carcinoma [28,49], its role in PC remains poorly defined. Additional studies will be required to elucidate the mechanistic and biological roles of *TNFRSF1B*, *NFKBIZ*, and *NFKBIL1* in PC.

In this context, the interrogation of publicly available IHC data from the HPA provided preliminary protein-level context for these transcriptional observations, indicating that the grade-associated differences identified at the transcriptomic level may also be reflected at the protein level. The observed staining patterns were broadly consistent with these trends; however, given the qualitative nature of HPA data and the absence of cohort-specific quantitative protein analysis, these findings should be interpreted as supportive rather than confirmatory. Quantitative validation using IHC on independent cohorts is warranted to confirm these expression patterns.

It is important to consider differences between the present cohort and publicly available datasets used for comparison, including variations in sample type (FFPE vs. fresh frozen), cohort composition, Gleason score stratification, and control selection (BPH vs. adjacent normal tissue). Despite these differences, both datasets capture grade-associated transcriptional variation in prostate adenocarcinoma, and the consistent expression trends observed for selected candidate genes support the robustness of the findings. However, these comparisons are best interpreted at the level of transcriptional concordance rather than direct cohort equivalence.

The proliferative phenotype of HG tumors was further supported by the significant upregulation of several Prolaris^®^ cell-cycle progression genes, including *TOP2A*, *BIRC5*, *CDCA8*, *FOXM1*, *PLK1*, *RRM2*, and *NUSAP1*, consistent with transcriptional features associated with aggressive biological behavior. Gene frequency prioritization further highlighted recurrent transcriptional patterns associated with proliferation and survival-related processes in HG tumors, including *AURKA*, *MYC*, and *PSMA1*, alongside the reduced expression of tumor suppressors such as *PTEN*, *PIK3R1*, and *CHD1*.

Although *AURKA* is well established in cell-cycle regulation, it has also been shown to support tumor survival through the stabilization of *BIRC5* via the *AURKA*–Survivin axis in gastric cancer [66]. While *AURKA* has been implicated in PC progression [67,68], its potential role in apoptosis regulation via Survivin stabilization remains to be clarified in this context. The concurrent upregulation of *AURKA* and *BIRC5* observed in this study is consistent with a transcriptional pattern associated with reduced apoptotic signaling; however, functional validation is required. Similarly, increased expression of *PSMA1* may contribute to proliferative and survival-associated transcriptional programs, as suggested in other solid tumors [51,69].

CIBERSORTx-based deconvolution suggested differences in inferred immune cell proportions; however, these estimates should be interpreted cautiously as computational inferences derived from bulk transcriptomic data. Analysis of immune cell composition provides an additional layer of biological insight into the transcriptomic changes observed across PC grades. LG tumors were enriched for immune cell populations associated with active immune surveillance, including resting CD4^+^ memory T cells, naïve B cells, follicular helper T cells, and differentiated macrophages (M1 and M2). This immune profile suggests a more immune-engaged TME, consistent with indolent clinical behavior and inflammatory signaling patterns observed in LG disease. Conversely, HG tumors demonstrated enrichment for monocytes and M0 macrophages, reflecting the accumulation of less differentiated myeloid populations associated with immune suppression or dysfunction. This shift in immune cell composition could contribute to PC progression by promoting a less immunologically responsive TME, although these interpretations remain inferential and require validation using higher-resolution approaches such as single-cell or spatial analysis.

Taken together, these findings suggest that Gleason grade progression is associated with coordinated transcriptional changes across proliferative, metabolic, and immune-related gene programs. Rather than reflecting direct pathway activation or suppression, the observed differences are more consistent with grade-associated transcriptional states and patterns of genes expression linked to tumor architecture and disease progression. The candidate genes identified in this study, including *SLC29A2*, *IL2RA*, *AAMDC*, *ASAH2*, *TNFRSF1B*, *NFKBIL1*, and *NFKBIZ*, provide a focused framework for future studies aimed at clarifying their biological and potential clinical relevance in PC.

This study has several limitations that should be acknowledged. First, the cohort size is relatively small, reflecting the exploratory nature of this pathology-based analysis, and therefore, the findings should be interpreted with appropriate caution. In addition, despite the pathological selection of samples with high tumor cellularity (>70%), residual tissue heterogeneity cannot be fully excluded. Also, bulk RNA sequencing captures averaged transcriptional signals across heterogeneous tissue samples and does not resolve cell-type-specific contributions within the TME. As a result, the observations reported here represent grade-associated transcriptional patterns rather than direct mechanistic evidence of pathway activity or cellular interactions. In addition, the availability of comprehensive clinical data for this cohort was limited, which restricted correlation with treatment outcomes, recurrence, and survival. Furthermore, protein-level validation was limited to the descriptive analysis of HPA data, and cohort-specific quantitative validation was not performed; therefore, validation using IHC is required to confirm these findings. Future studies incorporating larger cohorts, higher resolution approaches such as spatial transcriptomics or single-cell RNA sequencing, along with functional validation, will be essential to further dissect tumor heterogeneity, characterize microenvironmental interactions, and clarify the biological roles of the identified candidate genes. In addition, future studies using well-annotated cohorts with comprehensive clinical follow-up will be essential to further validate the prognostic and translational relevance of these observations.

## 5. Conclusions

In summary, this study provides a pathology-guided transcriptomic characterization of prostate cancer progression across Gleason grades in a MENA cohort. By integrating histopathological stratification with RNA sequencing and external cohort validation, we identified grade-associated transcription patterns that distinguish LG from HG disease and highlight candidate genes with potential clinical relevance, including SLC29A2, IL2RA, AAMDC, ASAH2, TNFRSF1B, NFKBIL1, and NFKBIZ.

Importantly, these findings should be interpreted as transcriptional associations linked to tumor grade rather than as direct mechanistic evidence. Within the context of the study design, the results provide biologically relevant insights into molecular changes associated with disease progression and establish a focused framework for future investigation aimed at functional validation and biomarker development. Overall, this work contributes to a growing understanding of PC heterogeneity and supports the value of pathology-informed transcriptomic profiling in refining disease characterization. Further validation in larger, independent cohorts and functional studies will be essential to confirm the clinical and biological relevance of the identified candidates.

## Figures and Tables

**Table 1:** Primers sets used for qRT-PCR.

Gene	Forward Primer (5′~3′)	Reverse Primer (5′~3′)
*SLC29A2*	TCCTGGGAAGTGGAGTCAGT	CAGCCAGTCCATGATGTTGAA
*AAMDC*	CTTGGGATTGGAGAGAAACAGG	GAGTCTGTACACCCTTCTCAAC
*ASAH2*	CATGGTATCACAAAGGCTCAGG	CTCTTCTGTACAGGGAGCCATA
*TNFRSF1B*	CTCTGGAACTGGGTTCCCGAGT	CCGAGTGCAGGCTTGAGTTTC
*NFKBIZ*	AGACTGCTGGGGAAGAACAC	CTCCCTTCTGAATCGCCTGAA
*GAPDH*	TGTCAGTGGTGGACCTGACCT	TCGCTGTTGAAGTCAGACGAG

**Table 2:** Overview of average sequencing quality metrics obtained from the NGS experiment.

Group	NGS Output Metrices		
	Mean Read Length (bp)	Mean Mapped Reads (10^6^)	Mean Bases ≥ Q20 (10^8^)
**High Grade**	81	5.47	4.92
**Low Grade**	86	6.91	6.21
**Control**	81	4.52	3.73

## Data Availability

The RNA sequencing data used in this study have been deposited and are publicly available in the Gene Expression Omnibus repository (https://www.ncbi.nlm.nih.gov/geo/ (accessed on 20 January 2026)) under accession number GSE1389845 (BioProject—NCBI). All other data, including R scripts used in this study, are available upon request from the corresponding author.

## Supplementary Materials

The following supporting information can be downloaded at https://www.mdpi.com/article/10.3390/cancers18182898/s1; Table S1. Demographic and clinicopathological data; Table S2A. Differentially expressed genes in HG compared to control; Table S2B. Differentially expressed genes in LG compared to control; Table S3A. Shared DEGs across HG and LG compared to control; Table S3B. Shared upregulated DEGs across HG and LG compared to control; Table S4. The expression pattern of five consistently upregulated common genes; Table S5. Differentially expressed genes across HG compared to LG tumors; Table S6. The expressions pattern of prostate cancer related genes; Table S7A. Upregulated genes across HG compared to LG tumors; Table S7B. Downregulated genes across HG compared to LG tumors; Table S8. The enriched pathways identified form MsigDB collections including C2, C5 and C7; Table S9. Expression pattern of PI3K related genes; Table S10. Expression pattern of NFκB related genes; Table S11. Relative proportions of immune cell subsets estimated by CIBERSORTx. Figure S1. Volcano plot and heatmap of DEGs among LG vs. Ctrl and HG vs. Ctrl tumor comparisons. (A) Volcano plot showing the DEGs identified in the LG vs. control comparison. (B) Volcano plot showing the DEGs identified in the HG vs. control comparison. The *x*-axis represents the log2FC, and the *y*-axis represents the –log_10_ (*p*-value). Statistically significant upregulated genes (|log2FC| > 1 and *p* < 0.05) are represented in red, while statistically significant downregulated genes (|log2FC| < −1 and *p* < 0.05) are represented in blue. (C) Heatmap of the LG vs. control comparison using unsupervised hierarchical clustering. (D) Heatmap of the HG vs. control comparison using unsupervised hierarchical clustering. Control is annotated as Ctrl in the figure. Figure S2. Association between overall survival and candidate gene expression in PRAD. (A) High expression of SLC29A2 showed a statistically significant poor overall survival (*p* = 0.028), while (B) higher expression of IL2RA did not induce poor overall survival. Figure S3. Consistent elevated expression of SLC29A2 and IL2RA genes across prostate cancer grades. Boxplots showing the VST normalized expression of these genes in both comparisons. (A) Significantly elevated expression of IL2RA in LG (*p* = 0.0144) and (B) significantly elevated expression of IL2RA in HG (*p* = 0.0459), (C) significantly elevated expression of SLC29A2 in LG (*p* = 0.016), (D) significantly elevated expression of SLC29A2 in HG (*p* = 0.00072). Figure S4. Principal component analysis (PCA), volcano plot, and heatmap of DEGs among HG vs. LG tumor comparison. (A) Principal component analysis (PCA) showed a clear separation between Hg and LG tumors. (B) Volcano plot showing the DEGs identified in HG vs. LG comparison. The *x*-axis represents the log2FC, and the *y*-axis represents the –log_10_ (*p*-value). Statistically significant upregulated genes (log2FC > 1 and *p* < 0.05) are represented in red, while statistically significant downregulated genes (log2FC < −1 and *p* < 0.05) are represented in blue. (C) Heatmap of the HG vs. LG comparison using unsupervised hierarchical clustering. The black boxes represent the distinct clusters identified in both tumor grades. In the Top left (Cluster #1) represent genes highly expressed in LG tumors compared to HG tumors, while in the bottom right (Cluster #2), represent genes with increased expression in HG tumors compared to LG tumors. Figure S5. Selective enriched pathways from GSEA of C5 gene ontology collection. Enriched pathways include (A) negative regulation of programmed cell death and (B) regulation of growth. Heatmaps demonstrate the expression profiles of genes within representative pathways. Red color reflects high gene expression levels, while green color reflects low gene expression. Figure S6. Representative IHC images of candidate biomarker expression in prostate tissue obtained from “https://www.proteinatlas.org/humanproteome/tissue/prostate (accessed on 3 March 2026)”. IHC images retrieved from the Human Protein Atlas illustrating protein expression patterns across normal prostate tissue and LG and HG adenocarcinoma samples for (A) SLC29A2, (B) AAMDC, (C) ASAH2, (D) TNFRSF1B, and (E) NFKBIZ.

## Abbreviations

The following abbreviations are used in this manuscript:

absGSEA	Absolute gene set enrichment analysis
AMACR	Alpha-methylacyl-CoA racemase
ASAH1	Acid ceramidase
ASAH2	Neutral ceramidase 2
B3GAT1	Beta-1,3-glucuronyltransferase 1
BIRC5	Baculoviral IAP repeat-containing protein 5
BPH	Benign prostatic hyperplasia
CCL2	C–C motif chemokine ligand 2
CCL19	C–C motif chemokine ligand 19
CDCA8	Cell division cycle-associated protein 8
CHD1	Chromodomain helicase DNA-binding protein 1
Ctrl	Control
CXCL1	C–X–C motif chemokine ligand 1
CXCL13	C–X–C motif chemokine ligand 13
CXCL2	C–X–C motif chemokine ligand 2
CXCL6	C–X–C motif chemokine ligand 6
DEGs	Differentially expressed genes
DFS	Disease-free survival
FFPE	Formalin-fixed, paraffin-embedded
FOXM1	Forkhead box M1
GEPIA2	Gene Expression Profiling Interactive Analysis 2
HG	High-grade prostate cancer
HR	Hazard ratio
HSPA1A	Heat shock protein family A member 1A
IL1R1	Interleukin-1 receptor type 1
IL1R2	Interleukin-1 receptor type 2
IL2RA	Interleukin-2 receptor alpha
IRAK3	Interleukin-1 receptor-associated kinase 3
ISUP	International Society of Urological Pathology
LG	Low-grade prostate cancer
Log2FC	Log2 fold change
MYC	MYC proto-oncogene
NEK2	NIMA-related kinase 2
NFKB	Nuclear factor kappa B
NFKBIL1	Nuclear factor kappa B inhibitor-like 1
NFKBIZ	Nuclear factor kappa B inhibitor zeta
NIK	NF-κB-inducing kinase
NLRC5	NLR family CARD domain-containing 5
NUDT18	Nudix hydrolase 18
NUSAP1	Nucleolar and spindle-associated protein 1
OS	Overall survival
PCA	Principal component analysis
PC	Prostate cancer
PI3K-AKT	Phosphoinositide 3-kinase–AKT
mTOR	Mechanistic target of rapamycin signaling pathway
PIK3R1	Phosphoinositide-3-kinase regulatory subunit 1
PLK1	Polo-like kinase 1
PRAD	Prostate adenocarcinoma
PRDM2	PR domain-containing 2
PSA	Prostate-specific antigen
PSMA1	Proteasome subunit alpha 1
PTEN	Phosphatase and tensin homolog
RNA	Ribonucleic acid
RNA-seq	RNA sequencing
RIZ1	Retinoblastoma-interacting zinc finger protein 1
RRM2	Ribonucleotide reductase regulatory subunit M2
SLC29A2	Solute carrier family 29 member 2
TAB2	TAK1-binding protein 2
TAB3	TAK1-binding protein 3
TCGA	The Cancer Genome Atlas
TLR	Toll-like receptor
TNF-α	Tumor necrosis factor alpha
TNFRSF1B	Tumor necrosis factor receptor superfamily member 1B
TOP2A	DNA topoisomerase II alpha
UALCAN	University of Alabama at Birmingham Cancer data analysis portal
